# Impact of Duration of Farrowing Crate Closure on Physical Indicators of Sow Welfare and Piglet Mortality

**DOI:** 10.3390/ani11040969

**Published:** 2021-03-31

**Authors:** Maria Camila Ceballos, Karen Camille Rocha Góis, Thomas D. Parsons, Meghann Pierdon

**Affiliations:** 1 Department of Production Animal Health, Faculty of Veterinary Medicine—University of Calgary, Clinical Skills Building, 11877–85th Street NW, Calgary, AB T3R 1J3, Canada; 2 Swine Teaching and Research Center, New Bolton Center, Department of Clinical Studies, School of Veterinary Medicine, University of Pennsylvania, 382 West Street Road, Kennett Square, PA 19348, USA; karen.camille.zootecnista@gmail.com (K.C.R.G.); thd@vet.upenn.edu (T.D.P.); mpierdon@vet.upenn.edu (M.P.)

**Keywords:** farrow, housing, lactation, hinged farrowing crate

## Abstract

**Simple Summary:**

Many sows when lactating are housed in farrowing crates. This is to protect their piglets from being crushed when the sow lies down and is particularly vital in the first few days of the piglet’s life. There are many ways that crates can be configured, one that allows flexibility in considering the sow’s mobility and the safety of piglets is the hinged farrowing crate. This type of crate opens to allow the sow room to move around but can also be closed like a conventional farrowing crate. For farmers to use this crate, however, they need to understand when is the optimal time to open the crate that protects the piglet while allowing sows improved freedom of movement. In this study, we examined three groups, including one where the crate was always closed, a group where the crate was opened at four days after birth, and a group where the crate was opened at seven days of age. Our results indicate that opening the crates decreases sow’s risk for teat lesions, and crates can be opened at 7 days of age without increasing piglet mortality.

**Abstract:**

This study examines effects of opening hinged farrowing crates 4 or 7 days post-farrowing. Sows (*n* = 696) allocated to 3 treatments: PC—crate closed, T4—crate opened day 4, and T7—crate opened day 7 were assessed for body condition score (BCS), lameness, shoulder lesions and teat lesions. Piglet mortality was higher in T4 (27.8%) compared to T7 (23.9%) and PC (25.9%) *(p* < 0.05) which did not differ with T7 *(p* > 0.05). No difference in risk of being laid on was found 1–3 days post-farrowing with all crates closed *(p* > 0.05). Day 4–6, piglets in T4, experienced higher risk of being laid on compared to PC (IRR = 2.5, *p* < 0.05), and T7 (IRR = 2.3, *p* < 0.05). Days 7–15 post-farrowing, risk of piglets dying from being laid on was higher in open crates, T4 and T7, versus PC (T4: IRR = 3.89, T7: IRR = 3.5, *p* < 0.05). We found higher risk for teat lesions in PC sows at weaning (*p* < 0.05). With crates open, risk of piglets being laid on increased between 7 days and weaning, but total piglet mortality did not differ and the sow’s risk for teat lesions decreased. Our results, therefore, support opening crates at day 7.

## 1. Introduction

The farrowing crate was developed to reduce piglet losses and to facilitate human intervention by limiting sow’s movements during parturition and lactation [1,2]. Most piglets die early in life [3] by getting laid on under the sow [4,5]. As crushing can result in injuries and suffocation, pre-weaning mortality is both a production and welfare challenge. Farrowing crates are an attempt to balance the welfare of the piglets with the physical restriction of the sow. However, the mismatch between sow’s behavioral needs and the farrowing crate design results in a compromise to the welfare of the sow [6]. Additionally, there is increasing concern regarding the welfare of the sow during lactation [7,8].

There are approximately nine different options for housing a lactating sow and her piglets, which range from the conventional crate, hinged crate, to open pen and communal pen systems [9]. There are advantages and disadvantages to each of these housing systems concerning sow and piglet welfare during parturition and lactation [9]. In a hinged crate system, the sow is initially crated, but when the piglets reach a designated age, the crate is opened, providing the sow additional space. The hinged crate, though it, does not provide the same physical freedom to the sow that a farrowing pen provides, fits in a similar footprint to a standard farrowing crate and protects the piglets for a designated period of time early in life when mortality risk to the piglet is the greatest.

The hinged crate, therefore, may be a practical solution for farmers. Studies have reported that closed crate sows weaned more pigs than open crate sows [10,11]. However, Mack et al. [12] reported that hinged crates could yield similar pre-weaning mortality to a closed crate. Similarly, Moustsen et al. [13] observed no differences in mortality or litter size at weaning. Verhovsek et al. [10] opened the crates when the pigs were 2 days of age; Chidgey et al. [11] and Moustsen et al. [13] opened the crates at 4 days of age; and Mack et al. [12] opened them at 14 days of age. These results suggest that 2 days closed is not sufficient for piglet protection and that, although 4 days may be long enough, piglet mortality may be lower with longer crate closure duration. In order to extend the findings of previous work, the aim of this study was to examine the effect of opening a hinged farrowing crate 4 or 7 days post-farrowing on piglet mortality and physical indicators of sow welfare. We hypothesized that we would find no differences in total piglet mortality between treatments and that we would see improvement in the physical indicators of sow welfare when hinged crates were opened earlier in lactation.

## 2. Animals, Materials and Methods

### 2.1. Animals, Housing and Experimental Design

**Figure 1:**
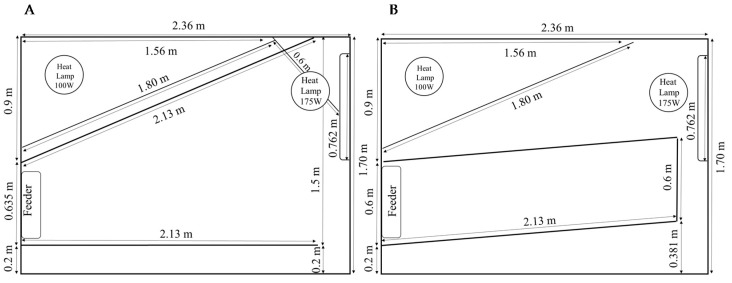
Schematic of pen equipped with a hinged farrowing crate. (**A**) Open. (**B**) Closed.

The study was conducted on a 5000-sow commercial farm located in the north central region of Pennsylvania, USA, between April and June 2019. A total of 696 sows, Line 200 or 241 (DNA Genetics, Columbus, NE, USA), with parity 2 ± 1.9 (range: 0–7), were initially included in the study. Sixty-six sows were removed from the study before completion due to illness or death. Prior to farrowing, sows were housed in large gestation pens, containing approximately 250 sows, where they were fed via electronic sow feeding (ESF). At approximately gestational day 113 (Loading day), sows were moved into a farrowing room. In total, six weekly farrowing cohorts of approximately 114 individuals were studied and housed in six identical rooms (35 × 20.5 m). Each room had 6 rows of 19 farrowing pens, or 114 pens per room, each of which was equipped with a hinged farrowing crate. The hinged crate could be opened to provide the sow additional space and freedom of movement, including being able to turn around. The farrowing pen dimensions were 2.36 × 1.70 m and provided 4.0 m^2^ in total area. The farrowing pen layout included a triangular creep area with a hover (0.9 × 1.56 × 1.8 m), and a farrowing crate with hinged sides with dimensions of 0.635 × 2.13 m located diagonally within pen (Figure 1). Both solid and partially open partitions were used as walls of the pens between sows. All sows and piglets were housed on perforated plastic flooring.

Upon entry to the farrowing rooms, sows were loaded into a hinged farrowing in the closed configuration. All sows were blocked by parity and randomly allotted to three treatment groups: PC—sow permanently crated until weaning (*n* = 177 sows), T4—sows crated until the fourth day post farrowing, when the crate was opened and remained so until weaning (*n* = 185 sows) and, T7—sows crated until the seventh day post farrowing, when the crate was opened and remained so until weaning (*n* = 161 sows) (Table 1). Crates were opened between 6:00–8:00 in the morning on a respective day for each treatment (fourth or seventh post farrowing).

Upon entry to the farrowing rooms, sows were fed once a day 4 pounds of a lactation diet that met or exceeded the National Research Council (NRC) guidelines (NRC, 2012). Two days post-farrowing, sows were started on ad libitum access to feed of up to 14.5 kg per day. The feed system was automatic, filling four times a day a tube with a capacity of 3.6 kg. The number of functional teats was determined for each sow prior to farrowing. Farm practice was to induce up to 20 sows per day to farrow if they reached day 116 of gestation without farrowing by giving a 5 mg injection of dinoprost tromethamine (Lutalyse^®^, Zoetis, Parsippany, NJ, USA). A total 179 sows or 29.7% of the study sows were induced to farrow.

The piglets received creep feed five days before weaning, at an average age of 19 ± 2 days. All sows and their litters had free access to water via nipple drinker (one for the sows and one for the piglets). The creep area was maintained at approximately 35 °C by a radiant heat lamp (100 W), and another lamp (175 W) was maintained in the back of the pen for the first three days post farrowing (prior to the opening of the hinged farrowing crate). No bedding or other substrate was provided in the farrowing area. Cross fostering of piglets between litters was carried out during the first 24 h post-farrowing if the number of live piglets farrowed by a sow exceeded her number of functional teats. All low viability and runt piglets were euthanized at this time. Baby pig processing, including castration, was completed by Day 3 post-farrowing. The same caretakers performed the handling of all study sows and litters. Including all sows involved in the study, 63 were early weaned for lactating poorly and recorded as a nurse off event (31, 10 and 22 from PC, T4 and T7, respectively). There were also 135 sows that had their lactation extended, a nurse on event, when they received piglets from a nurse off event (38, 39 and 58 from PC, T4 and T7, respectively).

### 2.2. Productivity Measures

A piglet was classified as having died from being laid on if they were found with their body flattened and cyanotic and the date of death was documented. Other causes of death including starvation, rupture, savaged, deformed, joint problem, lame, scrotal and belly rupture, blind anus, and no teats were also recorded. The sow’s parity and farrowing date were recorded for each dead piglet. For each litter, the number born alive, total born, total mummies, total weaned, weaning date, cross fostering events, nurse on events, and nurse off events were recorded. Percentage mortality was calculated as the number of piglets that died divided by the number of piglets in the litter on day 1 post standardization.

### 2.3. Sow Welfare Indicators

Assessment of sow physical indicators included the measurement of body condition score (BCS), lameness, shoulder lesion and teat lesions. BCS was evaluated using a standard visual scale based on the quantity of backfat and prominence of hipbones and spine using the following scale: 1: emaciated; 2: thin; 3: ideal; 4: fat; and 5: overly fat [14]. Lameness was scored as absence or presence of any sign of lameness, such as shifting the weight away from a limb and/or limping when walking. Shoulder lesion was scored with the following scale 1: the absence of a shoulder lesion; 2: the presence of a shoulder lesion (ulcerated dermis, sometimes covered with a scab) (adapted from Meyer et al. [15]). Teat lesions were quantified by counting the number of teats each sow had with either deep or superficial wounds. All indicators were measured on loading day and the day before weaning (on average 19 ± 3 days after farrowing).

### 2.4. Statistical Analysis

The total percent of piglet mortality was compared between treatments by fitting the data with generalized linear mixed models using PROC GLIMMIX in SAS, version 9.4 (SAS Institute Inc., Cary, NC, USA). All models included treatment (PC, T4 or T7), parity (0–7) and sow genetic line (241 or 200) as fixed effects. The interaction between parity and treatment was evaluated, and no interaction was found, so it was not included in the final model. Cohort (1–6) was included in the model as a random effect. Litter size after equalization (total born alive + foster in—foster off) was included as a covariate. Only data for sows that nursed their litter for at least 15 days after farrowing were included. From the original 630 study sows, 107 were nursed off before 15 days post-farrowing, or were used as nurse sows for other litters so after these exclusions, 523 sows were included.

Mixed effect Poisson regression models were used to assess the risk of piglet mortality for the recorded reasons including low viability, laid on, scours, and other reasons. Other included starvation, rupture, savaged, deformed, joint problem, lame, deformed, scrotal and belly rupture, blind anus, and no teats, which were combined as each category represented less than 5% of the mortalities. Poisson models included treatment, parity, and sow genetic line as fixed effects, cohort as random effects. Litter size after equalization was included as a covariate. Estimated coefficients were transformed and reported as incidence-rate ratios (IRR). A Bonferroni correction was used to account for multiple comparisons between the 3 treatments.

As one of the main concerns of using hinge farrowing crates is the mortality due to piglets being laid on, mixed effect Poisson regression models were used to assess the risk of piglets getting laid on in 3 different periods. These periods included day 0 to 3, where all treatments had the farrowing crates closed, day 4 to 6, where only the day 4 group (T4) had their crate opened, and days 7 to 15, where both the 7 day (T7) and 4 day group (T4) had their crates opened but before any litters had been early weaned (day 0 = day of birth). Poisson models included treatment (PC, T4 or T7), parity (0–7), and sow genetic line (241 or 200), as fixed effects, cohort (1–6) as random effects, and litter size after equalization as a covariate. Estimated coefficients were transformed and reported as an IRR. A Bonferroni correction was used to account for multiple comparisons between the 3 treatments.

For sow physical indicators, cohort was included as random effects in every model. Body condition score at weaning was analysed with a mixed effect ordinal regression model including treatment, parity, and sow genetic line, as fixed effects and body condition at loading into farrowing as a covariate. Coefficients for the ordinal model were transformed to IRR’s. Lameness at weaning was assessed with a mixed effect binomial regression model including treatment, parity, and sow genetic line as fixed effects, and lameness score at loading into farrowing as a covariate. Coefficients generated by the model were transformed to odds ratios. Whether a sow had a shoulder lesion at weaning was analysed using a mixed effect binomial model with treatment, parity, and sow genetic line as fixed effects, shoulder lesion score at loading into farrowing as a covariate, and coefficients were expressed as odds ratios. Number of lesioned teats at weaning was assessed using a mixed effect Poisson regression model with treatment, parity, and sow genetic line as fixed effects, and number of lesioned teats at loading into farrowing as a covariate. The Poisson model coefficients were transformed to incident rate ratios and the margins used to generate predicted counts. A Bonferroni correction was used to account for multiple comparisons between the 3 treatments.

Poisson, binomial, and ordinal modelling was done using STATA version 15 (StataCorp LP, College Station, TX, USA). All data was checked for normality using the Shapiro Wilk test. No outcomes were found to be normally distributed. The sow was the experimental unit for statistical analysis. A *p*-value of < 0.05 was treated as significant and a tendency when 0.05 < *p* < 0.10.

## 3. Results

### 3.1. Productivity

There was a significant effect of treatment on percentage of mortality (F_2, 449_ = 3.03, *p* = 0.04), with the highest percentage of mortality for T4 (27.8a ± 19), compared to T7 (23.9b ± 16; *p* = 0.04), and PC (25.9b ± 20; *p* = 0.03), which did not differ with T7 *(p* = 0.94) [real means ± SD]. The causes that contributed to total mortality included laid on (6.2, 8.5 and 7.2%), low viability (9.0, 7.9 and 7.6%), and scours (6.5, 6.1 and 4.5%), and other causes contributed less (4.2, 5.3 and 4.5%) for PC, T4 and T7, respectively. The number of weaned piglets per litter was 9.7 ± 2.7, 9.3 ± 2.2 and 9.8 ± 2.4 for PC, T4 and T7, respectively.

### 3.2. Risk of Piglet Death from Being Laid On

Overall, there was a significant effect of treatment on the risk of a piglet getting laid on *(p* = 0.04). There was no difference between T4 compared to T7 (IRR = 1.12; CI 95% 0.91 to 1.38; *p* = 0.30), neither between T7 compared to PC *(p* = 0.54), but there was a significant increase in risk for T4 compared to PC *(p* = 0.04), (Table 2).

For period 0–3 days when all crates were closed, no differences in the risk of being laid on were found when comparing T4 to PC (IRR = 0.83; CI 95% 0.64 to 1.08; *p* = 0.513) or T7 to PC (IRR = 0.92; CI 95% 0.70 to 1.20; *p* = 1.00) and no difference between T4 and T7 was found *(p* = 1.00).

For the period 4–6 days post-farrowing, T4 piglets were at higher risk for being laid on compared to PC (IRR = 2.5 ± 0.74, CI 95% = 1.37 to 4.43; *p* = 0.009) but not T7 (IRR = 2.3 ± 1.3, CI 95% = 0.72 to 7.43; *p* = 0.483). T7 had no significant difference with PC (IRR = 0.93 ± 0.35, CI 95% = 0.45 to 1.95; *p* = 1.00) for this period.

**Figure 2:**
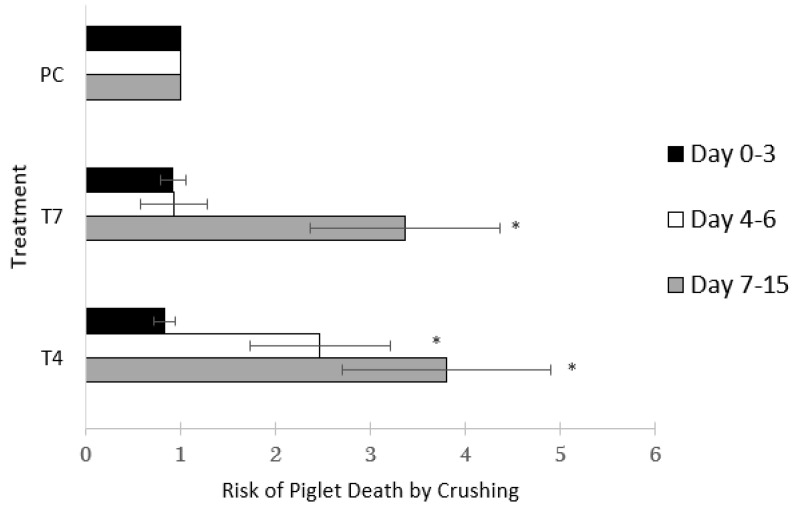
Effect of treatment on the relative risk (95% CI) of piglet death from being laid on, for three different periods: day 0 to 3, where all treatments had the farrowing crates closed, day 4 to 6, where the day 4 group had their crate opened, and days 7 to 15, where both the 7 day and 4 day group had their crates opened but before any litters had been early weaned. Treatment groups are denoted as PC, sow permanently crated until weaning, T4, sows crated until the fourth day post-farrowing, and T7, sows crated until the seventh day post-farrowing. Values marked by an asterisk are statistically different from PC group *(p* < 0.05) for that time period.

For period 7–15 days after birth, when T4 and T7 crates were open, the risk of piglets dying from being laid on was higher for T4 compared to PC (IRR = 3.80 ± 1.1; CI 95% = 2.16 to 6.7; *p* = 0.0001) and T7 (IRR = 12.8 ± 7.1; CI 95% = 4.30 to 37.95; *p* = 0.000001). T7 piglets also had significantly higher risk of being laid on compared to PC (IRR = 3.36 ± 1.0; CI 95% = 1.87 to 6.0; *p* = 0.0001) (Figure 2). Table 3 describes the magnitude of laid on mortality as a percentage of the mortality in different periods and demonstrates how this type of mortality is distributed across lactation. Laid on mortality is greatest during days 0–3.

### 3.3. Risk of Piglet Death for Low Viability, Scours, Other Reasons, and All Reasons

For piglet mortalities recorded as low viability, there was no effect of treatment *(p* = 0.95). There was no significant effect of treatment on the risk of piglets dying from scours *(p* > 0.05). For other causes, there was a significant effect of treatment *(p* = 0.03) with a trend for T4 to be at higher risk than PC *(p* = 0.08) but no significant difference between PC and T7 *(p* = 1.00) or T7 and T4 *(p* = 0.40). For mortality including all reasons there was a significant effect of treatment *(p* = 0.021) with T4 (IRR = 1.16 ± 0.07; CI 95% = 1.03 to 1.30) at higher risk of dying compared to T7 *(p* = 0.037) and a trend for T4 piglets (IRR = 1.13 ± 0.06; CI 95% = 1.01 to 1.26) to be more at risk than PC piglets *(p* = 0.084). There was no significant difference between T7 and PC *(p* = 1.00). Table 2 describes the effect of treatment on the risk of death for each recorded mortality reason.

### 3.4. Sow Welfare Indicators

Treatment had no effect on BCS *(p* = 0.46), lameness *(p* = 0.92), or shoulder lesions *(p* = 0.90) at weaning. On loading into farrowing accommodations, 1.90, 1.95 and 2.53% of the sows had a shoulder lesion, and at weaning day, this had increased to 25.0, 23.5 and 24.2% of sows with a shoulder lesion for PC, T4 and T7, respectively. The median ± IQR BCS for all treatments both at loading and weaning was 4.0 ± 0.0. The percentage of sows with lameness at loading day was 13.68, 12.20 and 9.55% for PC, T4 and T7, respectively and these percentages decreased at weaning day to 6.83, 5.78 and 5.37% for PC, T4 and T7, respectively.

Treatment significantly impacted the number of lesioned teats *(p* = 0.0011). T4 sows had fewer teat lesions (Predicted count = 0.37 + 0.06) compared to PC sows (Predicted Count = 0.63 + 0.09; *p* = 0.013), and there was a trend for a significant difference between the T7 group (Predicted Count = 0.43 + 0.08) and PC *(p* = 0.052). There was no difference between the T4 group and the T7 group *(p* = 1.00). Table 4 describes the effect of treatment on the risk of teat lesions and other sow indicators.

## 4. Discussion

Decreasing sow confinement during lactation allows her more freedom of movement, increases the availability to perform a diversity of behaviors [16] and improves sow welfare, as measured by different behavioral and physical welfare indicators [6]. However, the choice to eliminate confinement, or only temporarily confine a sow during lactation, has a potential influence on piglet welfare and survival. Here, we examined the impact of opening a hinged farrowing crate at 4 or 7 days post-farrowing on the total percentage of piglet mortality, the risk of piglet death due to crushing and other causes, and physical indicators of sow welfare. In the present study, litters were equalized shortly after birth based on individual sow functional teat counts and low viability and starve out pigs were promptly removed by barn staff for euthanasia in accordance with the pre-existing farm protocols. Consequently, all piglets that were evaluated in this study were viable and with no specific risk of dying due to being laid on.

We found that the total percentage of piglet mortality was higher for T4 litters than T7 and PC, and T7 did not differ from the PC group. This is consistent with studies that have found no differences in piglet survival when comparing loose housed sows with crated sows [5,17,18,19]. In contrast, a meta-analysis by Glencorse et al. [20], found that the risk of total piglet mortality was 14% higher for animals raised in pens compared with those in crates. Even with higher mortality, the same meta-analysis also found that there were no differences in the number of piglets weaned. The authors argue that most piglet mortality occurs before cross-fostering, a common husbandry technique adopted by commercial farms 24 h after farrowing, where dead piglets can be replaced. Spicer et al. [21] states that most piglet mortality occurs during the first 36 h post-farrowing, as we found in our study.

There are different causes of piglets’ mortality. KilBride et al. [5], in an epidemiological study in the United Kingdom involving 2826 piglets in different farrowing systems, found that crushing of healthy piglets was the most frequently reported cause of death, with 54.8%, and the subsequent causes were low viability (13.8%), starvation (6.8%), crushed while sick (4.7%) and diarrhea (3.5%). Considering the entire lactation period, we found that T4 presented a higher risk of piglet mortality caused by all reasons recorded in this study, and also for being laid on and for other reasons (including starvation, rupture, savaged, deformed, joint problem, lame, deformed, scrotal and belly rupture, blind anus, and no teats), compared to PC and T7. While no differences were found in the risk of piglets’ death due to low viability and scours between treatments, it is important to note that in the studied farm, there was a high percentage of euthanized piglets by the farm staff due to low viability and scours (15.5, 14.0, and 12.1% for T4, T7 and PC, respectively), accounting for almost or more than 50% of the total mortality for each treatment. The farm was experiencing a generalized scour problem, where almost all litters needed to be treated for scours in the first week of life. This specific situation at the farm explains the high total percentage mortality.

Farrowing crates are designed to protect piglets from death by being laid on from the sow, however, other studies have found different farrowing systems can impact other causes of piglet death during lactation. For example, Weber et al. [17] found significantly higher piglet mortality due to being laid on in loose farrowing pens, compared with crates, however the number of piglet deaths due to other reasons was higher in crates (including diarrhea, runts, bitten to death, etc.). The same tendency was found by KilBride et al. [5], where they conclude that farrowing crates reduce the risk of piglet mortality due to being laid on, compared to alternative farrowing systems (including crate/loose), however the risk of deaths due to other causes was increased in farrowing crates. Our results partially corroborated those studies with respect to T4. Related to scours, our results for both treatments (T4 and T7) agree with those of Chidgey et al. [11] who found no differences in deaths due to diarrhea between the standard crate and hinged crates opening them at 4 days post farrowing. Many studies evaluating the effect of farrowing systems on piglet mortality add together all the causes other than being laid on Weber et al. [18], KilBride et al. [5] and Olsson et al. [22]. It is difficult to find an explanation for the findings related to the mortality increasing due other reasons in T4, and further research should be done to see if there is any specific influence of the farrowing systems on the risk of death due to other reasons than piglets being laid on.

Considering the three different lactation periods proposed here to evaluate piglets mortality due to being laid on, one of the biggest concerns of farmers when using hinged farrowing crates, we found that the highest percentage of mortality was found in the first 3 days post farrowing (when all treatments had the crates in the closed position) and, as expected, no differences in the risk of piglets being laid on was found between treatments during this time period. Olsson et al. [22], working with temporary crates (opened at day 4) and loose crates, King et al. [23] also working with temporary crates but opening them at day 7, as well as Moustsen et al. [13] working with loose and temporary crates opening at 4 days and 7 days, demonstrated that the highest live-born piglet mortality occurred between 0 and 4 days. KilBride et al. [5] carried out an epidemiological study on 112 breeding pig farms in the UK comparing different farrowing systems (crate, crate/loose, loose, or outdoor) and found that, in the first 48 h post farrowing, the highest mortality was in crated and outdoor systems, compared with indoor loose and crate/loose systems. Pedersen et al. [18], comparing crates and loose pens, had the highest quantity of piglet deaths from being laid on occurred in the first 3 days of life. Nicolaisen et al. [24] found that most laid on events occurred during the first three days postpartum in loose housing pens and group-housing system, compared to the conventional crates. During this sensitive period, piglets are still gaining locomotor function, and understanding their environment. Nicolaisen et al. [24] evaluated piglet behavior during the first 3 days of life and found a reduced use of the creep area that left piglets in hazardous areas during the body posture changes of the sow. Our study is consistent with other studies indicating that reducing piglet mortality in the immediate post-partum period remains a challenge regardless of lactation housing type.

In the present study, mortality due to being laid on decreased from day 4 through the end of lactation (from almost 4.5% for the first 3 days to 2.81% from day 4 until weaning for all sows). KilBride et al. [5] found that the risk of death in piglets decreases as the piglets get older. However, even though mortality decreased as the piglets aged, there was a treatment effect on the risk of piglets being laid on within different time periods. After opening the crates, piglets from T4 and T7 had a higher risk of being laid on, compared with the closed crate litters. Previous studies have differing results on the risk of piglet death by being laid on when sow confinement is removed. Similar to our results, Chidgey et al. [11], comparing temporary crates (opening at day 4) with conventional farrowing crates, found that after opening the crate, a greater proportion of piglets died by being laid on. In contrast to our findings, other studies, such as [16,18,19,24] did not find a significant effect of housing on piglet mortality from being laid on.

Being laid on is typically one of the most common reasons for pre-weaning mortality, especially early in lactation. KilBride et al. [5] found that around 55% of the total mortality was caused by being laid on. In our study, the highest percentage of mortality due to being laid on was found for T4 (31%) followed by T7 (30%) and PC (24%). These proportions are even lower than the proportion of laid on piglets found in crated sows by Nicolaisen et al. [24] who reported a total of 36.1% of deaths from being laid on. Rosvold et al. [25] studied different management routines influencing piglets’ survival in loose-housed sow herds and identified the high degree of staff presence during farrowing as one of the important factors leading to reduced piglet mortality. Baxter et al. [26] mention that when judging any farrowing system, it must be emphasized that the quality of stockpeople handling and the maternal characteristics of the sow will be integral to its success. Husbandry practices on the study farm dictated systematic management routines during the first days of farrowing, but only one person was available to assist farrowing of two rooms of 114 sows, which could not be enough to assist all the sows as desired.

Differences between farm facilities, staffing, and husbandry practices likely can all contribute to inconsistencies in findings between different studies. Anti-crush embellishments such as sloping walls or bars in the farrowing pens are essential to reduce the risk of piglets being laid on [27]. The farm where this study was carried out had a pen design with anti-crush bars, to create space for the piglets to escape out from under a sow if they were trapped against a wall. It was not enough, however, to diminish the risk of piglets being laid on when the sow had total access to the pen space. The differences in the risk of being laid on could be influenced too by the individual maternal behavior expressed by sows [26], and also their lack of experience with this alternative farrowing system. King et al. [28] found that after the sows have experience with an alternative farrowing system, their piglet mortality reduces on subsequent farrowing. The sows of our study never experienced farrowing in this kind of crate, and the experience was novel for staff as well. The change for both the animals and stockpeople could have influenced our results.

We found no association between treatment and three of the sow physical welfare indicators evaluated in the study: BCS, lameness and shoulder lesions. The BCS of the sows at weaning was not influenced by treatment. Sows from the three treatments entered and left the farrowing room with 3.8 average BCS indicating that sows did not have problems with feed intake regardless of the crate position. On entering farrowing, 12% of the sows had lameness compared to 6% of the sows leaving the farrowing rooms, with no differences between treatments. Finally, 2% of the sows entered farrowing with shoulder lesions, and 24% of them had a shoulder lesions at weaning with no differences between treatments. Our results are similar to those of Meyer et al. [15] where the prevalence of shoulder lesions was 23% in sows defined as non-at-risk to develop this condition, after 3 weeks of lactation, in closed crates. Other studies have shown a lower prevalence of shoulder lesions (around 14%) in the same lactation time period [6,29], independent of the housing system where the sow was being kept. There are a lot of environmental and animal-related risk factors for shoulder lesions in sows [30] and the three most relevant sow-related risk factors are BCS, lameness, and parity [15]. Since we blocked by parity, and BCS and lameness were not different between treatments, our results agree with these previous studies where confinement duration is not a risk factor for shoulder lesions.

We found a higher risk for teats lesions in PC sows at weaning. Sows from PC presented 40% and 32% more lesioned teats compared to T4 and T7 sows, respectively. Our results confirm those found by other researchers who found a lower incidence of teat lesions in sows loose housed during lactation, compared to sows crated during lactation [6,10,31]). There are different possible explanations for these results. First, that sows cause abrasions to the teats with their hind limbs when getting up and lying down inside the closed crates, due to the restrictions imposed by the limited space allowed to them [10] and second, that in the confinement of the closed farrowing crate, sows are unable to avoid unwanted nursing events, compared to an open farrowing crate [6].

## 5. Conclusions

Even though the risk of crushing due to being laid on in different periods is higher for the opened farrowing crates, the total percentage of piglet mortality did not differ from the closed crate and the total risk of mortality was higher only for T4. This indicates that crating the sows immediately post-farrowing for seven days, when piglets are most vulnerable to crushing, has the potential to maintain a similar total percentage of piglet mortality, compared with crated conditions. Along with increased square footage allowed by the opened crate, we found an improvement in a physical welfare indicator in the hinged farrowing crates as we found a lower risk of teat lesions. Based on our results, the hinged farrowing crate should be considered. Combining our results and practical considerations related to piglet processing, castration, and vaccination strategies, which are easier in a closed crate, we suggest opening the crate at day 7 post-farrowing. Future studies focusing on sow maternal behavior and the best handling practices related to hinged farrowing crates should be researched.

## Figures and Tables

**Table 1:** Descriptive statistics for the number of sows and farrowing performance across treatment groups. All variables were collected prior to litter equalization on Day 1 and prior to treatment application on Day 4 or Day 7. Real means ± SD.

Variables	Treatment		
	PC ^1^	T4 ^2^	T7 ^3^
Sows (*n*)	177	185	161
Sow parity order	1.8 ± 1.8	1.9 ± 1.9	2.0 ± 1.9
Total born/litter (*n*)	16.3 ± 5.9	16.2 ± 6.8	14.7 ± 6.8
Live born/litter (*n*)	14.5 ± 3.4	14.4 ± 3.5	13.8 ± 3.9
Stillborn/litter (*n*)	1.1 ± 1.4	1.6 ± 2.4	1.2 ± 1.9

^1^ Sow permanently crated until weaning; ^2^ Sows crated until the fourth day post-farrowing; ^3^ Sows crated until the seventh day post-farrowing.

**Table 2:** Effect of treatment on the relative risk of piglet death for each recorded mortality reason from birth to weaning. Risk is denoted as incidence rate ratio (IRR) ± SE and 95% confidence interval (CI) for each treatment. *p*-values reflect the Bonferroni corrected values.

Mortality Reason	Treatment	IRR (SE)	CI (95%)	z	*p* > |z|
Laid on	T4 ^1^	1.30 ± 0.14	1.05 to 1.61	2.50	0.04
	T7 ^2^	1.17 ± 0.13	0.93 to 1.45	1.36	0.54
	PC ^3^	RC ^4^	RC	RC	RC
Low viability	T4	0.97 ± 0.09	0.80 to 1.16	−0.33	1.00
	T7	0.98 ± 0.09	0.81 to 1.20	−0.19	1.00
	PC	RC	RC	RC	RC
Scours	T4	1.05 ± 0.12	0.84 to 1.31	0.46	1.00
	T7	0.75 ± 0.10	0.58 to 0.96	−2.28	1.00
	PC	RC	RC	RC	RC
Other ^5^	T4	1.77 ± 0.40	1.14 to 2.76	2.54	0.08
	T7	1.25 ± 0.31	0.77 to 2.06	0.92	1.00
	PC	RC	RC	RC	RC
All	T4	1.13 ± 0.06	1.01 to 1.25	2.22	0.08
	T7	0.98 ± 0.06	0.87 to 1.10	−0.39	1.00
	PC	RC	RC	RC	RC

^1^ Sows crated until the fourth day post-farrowing; ^2^ Sows crated until the seventh day post-farrowing; ^3^ Sow permanently crated until weaning; ^4^ Reference class; ^5^ Includes starvation, rupture, savaged, deformed, joint problem, lame, scrotal and belly rupture, blind anus, and no teats.

**Table 3:** Percentage of piglet mortality (mean ± SD) due to being laid on across different periods post farrowing by treatment group.

Variables	Treatment		
	PC ^1^	T4 ^2^	T7 ^3^
Sows (*n*)	177	185	161
Total mortality (%) from being laid on			
0–3 days post-farrowing ^4^	4.99 ± 7.5	4.14 ± 6.9	4.24 ± 6.6
4–6 days post-farrowing ^5^	0.59 ± 1.9	1.59 ± 3.7	0.61 ± 2.3
7–15 days post-farrowing ^6^	0.57 ± 1.9	2.71 ± 5.4	2.37 ± 5.0

^1^ Sow permanently crated until weaning; ^2^ Sows crated until the fourth day post-farrowing; ^3^ Sows crated until the seventh day post-farrowing; ^4^ Where all treatments had the farrowing crates closed; ^5^ Where the day 4 group had their crate opened; ^6^ Where both the 7 day and 4 day group had their crates opened but before any litters had been early weaned.

**Table 4:** Body condition score (BCS), lameness and shoulder lesion odds ratio (OR) ± SE, incidence rate ratio (IRR) ± SE of teat lesions, and 95% confidence interval (CI) for each treatment at loading and at weaning.

Indicator	Treatment	OR (SE)	CI (95%)	z	*p* > |z| ^5^
BCS	T4 ^1^	1.34 ± 0.46	0.69 to 2.61	0.87	1.00
	T7 ^2^	1.52 ± 0.55	0.76 to 3.08	1.18	0.71
	PC ^3^	RC ^4^	RC	RC	RC
Lameness	T4	0.86 ± 0.42	0.33 to 2.22	−0.32	1.00
	T7	0.82 ± 0.44	0.32 to 2.34	−0.37	1.00
	PC	RC	RC	RC	RC
Shoulder Lesion	T4	0.89 ± 0.24	0.53 to 1.51	−0.43	1.00
	T7	0.90 ± 0.25	0.52 to 1.56	−0.46	1.00
	PC	RC	RC	RC	RC
**Indicator**	**Treatment**	**IRR (SE)**	**CI (95%)**	**z**	***p* > |z|**
Teat lesions	T4	0.61 ± 0.09	0.45 to 0.81	−3.42	0.013
	T7	0.68 ± 0.10	0.51 to 0.91	−2.64	0.052
	PC	RC	RC	RC	RC

^1^ Sows crated until the fourth day post-farrowing; ^2^ Sows crated until the seventh day post-farrowing; ^3^ Sow permanently crated until weaning; ^4^ Reference class; ^5^ *p*-Values reflect the Bonferroni corrected values.

## Data Availability

Not applicable.

## References

[1] Robertson J.B., Laird R., Hall J.K.S., Forsyth R.J., Thomson J.M., Walker-Love J.A. (1966). Comparison of Two Indoor Farrowing Systems for Sows. Anim. Sci..

[2] Edwards S.A., Fraser D. (1997). Housing Systems for Farrowing and Lactation. Pig. J..

[3] Hellbrügge B., Tölle K.H., Bennewitz J., Henze C., Presuhn U., Krieter J. (2008). Genetic Aspects Regarding Piglet Losses and the Maternal Behaviour of Sows. Part 2. Genetic Relationship between Maternal Behaviour in Sows and Piglet Mortality. Animal.

[4] Marchant J.N., Rudd A.R., Mendl M.T., Broom D.M., Meredith M.J., Corning S., Simmins P.H. (2000). Timing and Causes of Piglet Mortality in Alternative and Conventional Farrowing Systems. Vet. Rec..

[5] KilBride A.L., Mendl M., Statham P., Held S., Harris M., Cooper S., Green L.E. (2012). A Cohort Study of Preweaning Piglet Mortality and Farrowing Accommodation on 112 Commercial Pig Farms in England. Prev. Vet. Med..

[6] Ceballos M.C., Góis K.C.R., Parsons T.D. (2020). The Opening of a Hinged Farrowing Crate Improves Lactating Sows’ Welfare. Appl. Anim. Behav. Sci..

[7] Council Directive 2008/120/EC of 18 December 2008 Laying down Minimum Standards for the Protection of Pigs (Codified Version). https://eur-lex.europa.eu/legal-content/EN/TXT/PDF/?uri=CELEX:32008L0120&from=EN.

[8] Baxter E.M., Andersen I.L., Edwards S.A. (2017). Sow welfare in the farrowing crate and alternatives. Advances in Pig Welfare.

[9] Johnson A.K., Marchant-Forde J.N. (2008). Welfare of Pigs in the Farrowing Environment. The Welfare of Pigs.

[10] Verhovsek D., Troxler J., Baumgartner J. (2007). Peripartal Behaviour and Teat Lesions of Sows in Farrowing Crates and in a Loose-Housing System. Anim. Welf..

[11] Chidgey K.L., Morel P.C.H., Stafford K.J., Barugh I.W. (2015). Sow and Piglet Productivity and Sow Reproductive Performance in Farrowing Pens with Temporary Crating or Farrowing Crates on a Commercial New Zealand Pig Farm. Livest. Sci..

[12] Mack L.A., Rossini S.P., Leventhal S.J., Parsons T.D. (2017). Case study: Differences in Social Behaviors and Mortality among Piglets Housed in Alternative Lactational Systems. Prof. Anim. Sci..

[13] Moustsen V.A., Hales J., Lahrmann H.P., Weber P.M., Hansen C.F. (2013). Confinement of Lactating Sows in Crates for 4 Days after Farrowing Reduces Piglet Mortality. Animal.

[14] Coffey R.D., Parker G.R., Laurent K.M. (1999). Assessing Sow Body Condition.

[15] Meyer D., Vogel C., Kreienbrock L., Große Beilage E. (2019). How Effective Are Clinical Pre-Farrowing Risk Assessment and the Use of Soft Rubber Mats in Preventing Shoulder Ulcers in at-Risk Sows?. Porc. Health Manag..

[16] Singh C., Verdon M., Cronin G.M., Hemsworth P.H. (2017). The Behaviour and Welfare of Sows and Piglets in Farrowing Crates or Lactation Pens. Animal.

[17] Weber R., Keil N.M., Fehr M., Horat R. (2007). Piglet Mortality on Farms Using Farrowing Systems with or without Crates. Anim. Welf..

[18] Pedersen L.J., Berg P., Jørgensen G., Andersen I.L. (2011). Neonatal Piglet Traits of Importance for Survival in Crates and Indoor Pens. J. Anim. Sci..

[19] Zhang X., Li C., Hao Y., Gu X. (2020). Effects of Different Farrowing Environments on the Behavior of Sows and Piglets. Animals.

[20] Glencorse D., Plush K., Hazel S., D’Souza D., Hebart M. (2019). Impact of Non-Confinement Accommodation on Farrowing Performance: A Systematic Review and Meta-Analysis of Farrowing Crates Versus Pens. Animals.

[21] Spicer E.M., Driesen S.J., Fahy V.A., Horton B.J., Sims L.D., Jones R.T., Cutler R.S., Prime R.W. (1986). Causes of Preweaning Mortality on a Large Intensive Piggery. Aust. Vet. J..

[22] Olsson A.-C., Botermans J., Englund J.-E. (2018). Piglet Mortality—A Parallel Comparison between Loose-Housed and Temporarily Confined Farrowing Sows in the Same Herd. Acta Agric. Scand. A Anim. Sci..

[23] King R.L., Baxter E.M., Matheson S.M., Edwards S.A. (2019). Temporary Crate Opening Procedure Affects Immediate Post-Opening Piglet Mortality and Sow Behaviour. Animal.

[24] Nicolaisen T., Lühken E., Volkmann N., Rohn K., Kemper N., Fels M. (2019). The Effect of Sows’ and Piglets’ Behavioura on Piglet Crushing Patterns in Two Different Farrowing Pen Systems. Animals.

[25] Rosvold E.M., Kielland C., Ocepek M., Framstad T., Fredriksen B., Andersen-Ranberg I., Næss G., Andersen I.L. (2017). Management Routines Influencing Piglet Survival in Loose-Housed Sow Herds. Livest. Sci..

[26] Baxter E.M., Lawrence A.B., Edwards S.A. (2012). Alternative Farrowing Accommodation: Welfare and Economic Aspects of Existing Farrowing and Lactation Systems for Pigs. Animal.

[27] Damm B.I., Forkman B., Pedersen L.J. (2005). Lying down and Rolling Behaviour in Sows in Relation to Piglet Crushing. Appl. Anim. Behav. Sci..

[28] King R.L., Baxter E.M., Matheson S.M., Edwards S.A. (2019). Consistency Is Key: Interactions of Current and Previous Farrowing System on Litter Size and Piglet Mortality. Animal.

[29] Lambertz C., Petig M., Elkmann A., Gauly M. (2015). Confinement of Sows for Different Periods during Lactation: Effects on Behaviour and Lesions of Sows and Performance of Piglets. Animal.

[30] Rioja-Lang F.C., Seddon Y.M., Brown J.A. (2018). Shoulder Lesions in Sows: A Review of Their Causes, Prevention, and Treatment. J. Swine Health Prod..

[31] Lohmeier R.Y., Gimberg-Henrici C.G.E., Burfeind O., Krieter J. (2019). Suckling Behaviour and Health Parameters of Sows and Piglets in Free-Farrowing Pens. Appl. Anim. Behav. Sci..

